# The Charlotte Project: Recommendations for patient-reported outcomes and clinical parameters in Dravet syndrome through a qualitative and Delphi consensus study

**DOI:** 10.3389/fneur.2022.975034

**Published:** 2022-09-01

**Authors:** Ángel Aledo-Serrano, Ana Mingorance, Vicente Villanueva, Juan José García-Peñas, Antonio Gil-Nagel, Susana Boronat, JoséÁngel Aibar, Silvia Cámara, María José Yániz, Luis Miguel Aras, Bárbara Blanco, Rocío Sánchez-Carpintero

**Affiliations:** ^1^Epilepsy Program, Neurology Department, Ruber Internacional Hospital, Madrid, Spain; ^2^Dracaena Consulting SL, Loulou Foundation, London, United Kingdom; ^3^Hospital Universitario y Politécnico La Fe, Valencia, Spain; ^4^Hospital Infantil Universitario Niño Jesús, Madrid, Spain; ^5^Hospital de la Santa Creu i Sant Pau, Barcelona, Spain; ^6^Fundación Síndrome de Dravet, Madrid, Spain; ^7^Clínica Universidad Navarra, Pamplona, Spain; ^8^Apoyo Dravet, San Sebastian, Spain; ^9^Hospital Universitario Virgen del Rocío, Seville, Spain

**Keywords:** neurodevelopment, developmental and epileptic encephalopathies, epilepsy, genetic epilepsy, caregivers, patient-reported outcomes, *SCN1A*

## Abstract

**Objective:**

The appropriate management of patients with Dravet Syndrome (DS) is challenging, given the severity of symptoms and the burden of the disease for patients and caregivers. This study aimed to identify, through a qualitative methodology and a Delphi consensus-driven process, a set of recommendations for the management of DS to guide clinicians in the assessment of the clinical condition and quality of life (QoL) of DS patients, with a special focus on patient- and caregiver-reported outcomes (PROs).

**Methods:**

This study was conducted in five phases, led by a multidisciplinary scientific committee (SC) including pediatric neurologists, epileptologists, a neuropsychologist, an epilepsy nurse, and members of DS patient advocates. In phases 1 and 2, a questionnaire related to patients' QoL was prepared and answered by caregivers and the SC. In phase 3, the SC generated, based on these answers and on a focus group discussion, a 70-item Delphi questionnaire, covering six topic categories on a nine-point Likert scale. In phase 4, 32 panelists, from different Spanish institutions and with a multidisciplinary background, answered the questionnaire. Consensus was obtained and defined as strong or moderate if ≥80% and 67–79% of panelists, respectively, rated the statement with ≥7. Phase 5 consisted of the preparation of the manuscript.

**Results:**

The panelists agreed on a total of 69 items (98.6%), 54 (77.14%), and 15 (21.43%) with strong and moderate consensus, respectively. The experts' recommendations included the need for frequent assessment of patient and caregivers QoL parameters. The experts agreed that QoL should be assessed through specific questionnaires covering different domains. Likewise, the results showed consensus regarding the regular evaluation of several clinical parameters related to neurodevelopment, attention, behavior, other comorbidities, and sudden unexpected death in epilepsy (SUDEP). A consensus was also reached on the instruments, specific parameters, and caregivers' education in the routine clinical management of patients with DS.

**Conclusions:**

This consensus resulted in a set of recommendations for the assessment of clinical and QoL parameters, including PROs, related to the general evaluation of QoL, neurodevelopment, attention, behavior, other comorbidities affecting QoL, SUDEP, and QoL of caregivers/relatives and patients with DS.

## Introduction

Dravet syndrome (DS) is a life-threatening epilepsy syndrome that begins in infancy or early childhood and includes a wide spectrum of symptoms ranging from mild to severe (1). Additionally, DS is included in the group of developmental and epileptic encephalopathies (DEE). Patients with DS initially present with prolonged focal or generalized onset motor seizures, which are usually fever-induced, starting before 15 months of age (often during the first year) (2). As the disease progresses, DS patients develop other symptoms, including neurodevelopmental impairments (3). Up to 88% of DS patients have mutations in the *SCN1A* gene (4), which encodes a sodium channel involved in nervous system function. Pathogenic variants of the *SCN1A* gene result in a wide range of disease severities, from severe DS, on one end of the spectrum, to milder pathologies on the other end, such as genetic epilepsy with febrile seizures plus (GEFS+) and the genetic syndrome with febrile seizures (FS or FS+) (5–8).

Dravet syndrome, first described in 1978 (1), has an estimated incidence of 1/12,000 to 1/40,000 live births (9, 10), although there is still a diagnostic gap, especially in adult patients. Children with DS experience significant developmental delays associated with behavioral problems, which become particularly apparent in the second to fourth years of life (11, 12). Even though these symptoms become more stable after adolescence, they persist throughout adulthood, impairing patients' quality of life (QoL) (12). Furthermore, the severity of these cognitive and behavioral problems cause psychosocial sequelae in the short and long term, requiring extensive care from caregivers and relatives (1, 13–15). DS patients experience fine and gross motor skill impairments and other physical disorders, including ataxia and gait disturbances (1, 16). Due to the severity and the burden of the disease, the quantity and quality of the support required from caregivers and relatives can be emotionally and financially challenging, resulting in a significant financial impact for families (17, 18). Furthermore, mortality rates in DS are high and, in 59% of the cases, they are due to Sudden Unexpected Death in Epilepsy (SUDEP) (19). Accordingly, experts acknowledge the need to assess different aspects of DS beyond seizures in the clinical management of DS, including the impact of DS on caregivers' and relatives' QoL (14). In this regard, patient-reported outcome measures (PROMs) are an increasingly used tool that has proven useful in various pathologies (20), but there is still little evidence in the context of patients with DS (21).

Several guidelines and recommendations on DS are available, including a recently published European treatment guideline (22). However, owing to the complexity of DS involvement and the significant uncertainty associated with its clinical assessment (23–27), there is still a need for instruments assessing the relevant aspects of DS for patients follow-up in the routine clinical practice, such as PROMs. Similarly, there is limited literature summarizing recommendations regarding the comorbidities of DS from different perspectives, such as professionals from multiple disciplines and caregivers. This study aimed to identify, through a qualitative focus group including patients and caregivers and a Delphi consensus-driven process, a core set of recommendations for the management of DS to guide clinicians in the evaluation of both the clinical condition and QoL of DS patients, with a special focus on patient/caregiver reported outcomes. Recommendations regarding the assessment of the QoL of caregivers and relatives of patients with DS were a secondary objective.

## Materials and methods

### Study design

The Delphi technique is a structured method broadly used to collect relevant information on a specific issue and consists of a series of questionnaires or “rounds” targeted to experts (28). The key features of this method are participant anonymity and controlled feedback. The Charlotte Project was carried out in five phases. In phases 1 and 2, a questionnaire assessing different QoL aspects was prepared. In Phase 2, caregivers answered the questionnaire and the results were discussed using a qualitative methodology to collect caregivers' perspectives. Patient characteristics and relationship with participating caregivers are shown in Supplementary Table 1. The scientific committee also answered the questionnaire. During phase 3, the Delphi statements were developed based on the results of the previous phases. In phases 4 and 5, the Delphi questionnaire was answered by a panel of experts, results were analyzed, and the manuscript was prepared.

### Study phases

**Figure 1:**
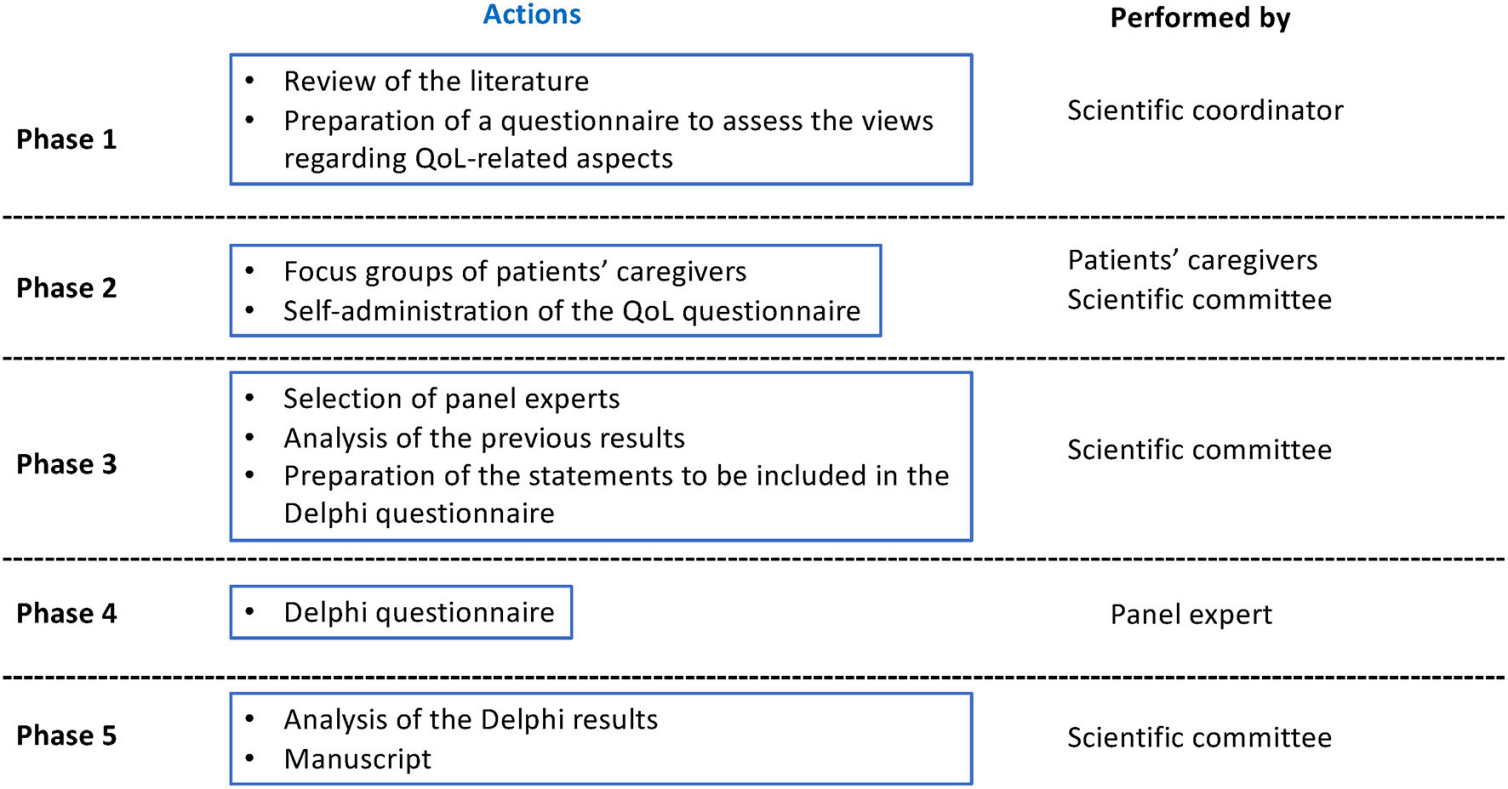
Study phase diagram.

A diagram of the study phases is presented in Figure 1. In the initial phase, conducted between March and April 2021, the literature regarding the assessment of QoL, comorbidities, and patient-reported outcomes measures (PROMs) in DS and other related DEEs was reviewed. Keywords searched (in English language) in the literature databases PUBMED and EMBASE in March–April 2021 (last 10 years period) included “Dravet Syndrome,” “Patient-reported outcome,” “Quality of life,” “Comorbidity,” and “Caregiver”. Also, during this phase, an *ad hoc* questionnaire was designed for this project to assess the views of the patients and the scientific committee regarding aspects related to QoL, the impact of comorbidities on QoL, and caregivers' QoL. The coordinator of the project (Dr. Aledo) was in charge of designing the questionnaire. In the beginning, the QoL-related publications found by the literature search were examined. Relying on literature and clinical experience, the questions were developed (Supplementary Table 2). In the second phase, the qualitative phase completed between May and June 2021, representatives of the Dravet Syndrome Foundation (DSF) Spain contacted caregivers (i.e., patients' relatives) and invited them to participate in a meeting. During the meeting, the eight invited caregivers answered the questionnaire and discussed relevant issues in a focus group. Additionally, caregivers of DS patients shared their opinions and experiences related to the care and management of the disease from physical, social, and emotional perspectives. The 10 members of the scientific committee also answered the questionnaire individually. During the third phase, the scientific committee compared, analyzed, and discussed the answers provided both by patients and the members of the scientific committee. The conclusions obtained during the meeting were used to prepare the statements to be included in the Delphi questionnaire. The final version of the questionnaire included 70 items written as statements to be answered on a nine-point Likert scale, where 1 was totally disagree and 9 totally agree. Additionally, the scientific committee selected the expert panel to participate in the Delphi phase. The fourth phase was the Delphi phase, conducted between September and October 2021, in which the panelists answered the questionnaire in one round. A free text space was also included to allow participants to provide additional comments for each item. A second round was not considered necessary, due to the high degree of consensus reached. In the fifth and final phase (November 2021), the manuscript was prepared.

### Scientific committee

The project was led by a scientific committee that was comprised of a multidisciplinary team with seven experts in the field of DS, including three pediatric neurologists, three epileptologists (both pediatric and adult), one neuropsychologist, one epilepsy nurse, one neuroscientist specialized in DEEs and working with patient organizations, and two members of DS patient advocates.

### Expert panel

The scientific committee selected a group of 32 epilepsy experts with a multidisciplinary background as participants (Supplementary Figure 1). The criteria for their selection included professional knowledge and experience in the field of DS. All participants were members of the Spanish Epilepsy Society, as well as the Epilepsy section of the Spanish Society of Pediatric Neurology or the Spanish Society of Neurology.

### Consensus definition

An item or statement was considered consensual when there was “agreement” of opinion in the panel. This happens when the panelists' votes outside one of the three-point regions [(1–3), (4–6), (7–9)] containing the median were less than one-third of the answers (<33.3%). The median value determines the group consensus: majority “disagreement” if the median was within 1–3 and majority “agreement” if the median was within 7–9. Cases where the median was within the 4–6 region were considered “doubtful”. Conversely, “discordance” was considered when the scores of one-third or more of the panelists were in the (1–3) region and another third or more in the (7–9) region. The remaining statements for which no concordance or discordance was obtained were considered to have an “undetermined” level of consensus. A strong consensus was defined if 80% or more of panelists providing an opinion rated the statement with 7 or higher. A moderate consensus was considered if 67–79% of panelists rated the statement with 7 or higher (29). Statements that did not reach this level of agreement were interpreted as “undetermined”.

### Data analysis

SPSS Statistics version 20 by IBM (Armonk, NY, USA) was used to create and analyze the database. The median and the percentage of responses in the 7–9 range were calculated, and their values were used to define consensus.

### Ethical aspects

This study was performed following the Helsinki Declaration. Data from the Delphi questionnaire were anonymized for the analysis. All the personal data were dissociated from the results in compliance with the EU General Data Protection Regulation (GDPR).

## Results

The 70 statements developed by the scientific committee covered a total of six categories: (1) general aspects of the QoL evaluation of patients with DS (15 items), (2) evaluation of neurodevelopment (24 items), (3) assessment of attention and behavior (9 items), (4) evaluation of other comorbidities affecting QoL (14 items), (5) Sudden unexpected death in epilepsy (SUDEP) (4 items), and (6) assessment of the QoL of caregivers/relatives (3 items). Of the 32 DS experts invited to participate in the Delphi process, a total of 28 (87.5%) answered the questionnaire (Supplementary Table 3). The panel experts reached a consensus on “agreement” in the first round on a total of 69 of the 70 items (98.57%). A strong consensus was obtained on 54 items (77.14%) and a moderate consensus was reached on 15 items (21.43%). Key results for each category are detailed below.

### General aspects of the evaluation of the QoL of patients with DS

A 100% consensus was obtained across category 1 with an agreement on all the 15 statements, although 4 (26.7%) were categorized into the moderate consensus range (Table 1). The panelists agreed that the evaluation of the QoL of the patient at each visit with the specialist was important. Self-administered structured questionnaires for relatives or caregivers in an electronic format was the recommended method for evaluating QoL.

**Table 1:** General aspects on the assessment of the quality of life of patients with Dravet syndrome (block 1).

**Statement**		**Median**	**% Consensus**	**Result**	**Consensus level**
1.1	It is recommended to evaluate the quality of life of the patient to determine the impact of the disease on their physical, mental and social wellbeing, facilitating follow-up and decision-making by physicians	9	100	Agreement	Strong
1.2	At each visit with the specialist, it is recommended to assess the quality of life of the patient taking into account the patient's biological or chronological age, according to the characteristics to be evaluated	8	75.0	Agreement	Moderate
1.3	It is recommended to assess the quality of life of the patient through specific questionnaires aimed at the parents or caregivers, taking into account the patient's biological or chronological age, according to the characteristics to be evaluated	8	78.6	Agreement	Moderate
1.4	In assessing quality of life, caregivers and family members should have tools such as self-administered questionnaires to be able to report the patient's situation to the specialist in a structured and concrete way	8	85.7	Agreement	Strong
1.5	The quality of life questionnaires used to monitor the patient should include specific questions on: Aspects related to physical health status	8	100	Agreement	Strong
1.6	The quality of life questionnaires used to monitor the patient should include specific questions on: Aspects related to the psychological status	9	96.4	Agreement	Strong
1.7	The quality of life questionnaires used to monitor the patient should include specific questions on: Aspects related to the social environment and functioning, such as the family or school environment	9	96.4	Agreement	Strong
1.8	The quality of life questionnaires used to monitor the patient should include specific questions on: Aspects related to the financial, housing conditions and supporting material	7	75.1	Agreement	Moderate
1.9	The quality of life questionnaires used for patient follow-up should include specific questions on: Aspects related to adverse effects of drug treatment	9	96.4	Agreement	Strong
1.10	The quality of life questionnaires used to monitor the patient should include specific questions on: Aspects related to the relationship of the patient and caregivers with healthcare professionals	7	82.1	Agreement	Strong
1.11	The quality of life questionnaires used to monitor the patient should include specific questions on: Aspects related to the relationship of the patient and caregivers with the healthcare system	7	82.1	Agreement	Strong
1.12	The electronic format is considered the optimal method for collecting information on quality of life (with different alternatives for non-technology users, such as paper format)	8	82.2	Agreement	Strong
1.13	The pediatrician, family doctor, pediatric neurologist, or neurologist should be the one to assess the results of the questionnaires on quality of life provided and to ask for them regularly and specifically in their visits and consultations	7	67.9	Agreement	Moderate
1.14	Patient quality of life questionnaires should always be provided and managed by healthcare professionals, with specialized nursing personnel, at the hospital or in a primary care center being the most recommended setting	8	85.7	Agreement	Strong
1.15	It is recommended to monitor and maintain fluent and constant communication between the different professionals involved in the quality of life of the patient, their family members, and caregivers	9	100	Agreement	Strong

Grey emphasis is used to highlights statements where there was no agreement or agreement was only moderate.

Regarding the content of the QoL questionnaires used to monitor patients, the experts reached a consensus that these tools should be specific to the syndrome and should consider the biological or chronological age of the patients. The questionnaires should include aspects related to the patient's physical health, the psychological status, the social environment and functioning, the economic situation, housing conditions and supporting material, adverse effects of pharmacological treatment, and the relationships of the patient and caregivers with healthcare professionals and the healthcare system.

To conclude this category, the panelists reached a consensus agreement regarding the administration and assessment of the QoL questionnaire. The experts stated that an electronic format is an optimal method and that the questionnaire should be administered and evaluated by healthcare professionals, always in communication and coordination with each other and with the patient's family and caregivers.

### Evaluation of neurodevelopment in patients with DS

The panelists reached a consensus agreement on all 24 statements (100%) of category 2, of which 6 (25.0%) had a moderate consensus (Table 2). In their opinion, a neurodevelopmental evaluation of DS patients is recommended at each visit with the specialist, at different frequencies depending on the age of the patient. The panelists agreed that the evaluation of various neurological parameters related to patient care and communication skills should be carried out at least every 3–6 months.

**Table 2:** Evaluation of neurodevelopment in patients with Dravet syndrome (block 2).

**Statement**		**Median**	**% Consensus**	**Result**	**Consensus level**
2.1	It is recommended to assess the neurodevelopment of patients with Dravet syndrome at each visit with the specialist (every 3 months from 0 to 3 years, every 6 months from 3 to 6 years, and every 12 months from 7 years onwards)	9	82.2	Agreement	Strong
2.2	It is recommended for all patients with Dravet syndrome to be assessed and monitored by a multidisciplinary team of neuropsychologists, speech therapists, occupational therapists and physiotherapists, grouping specialists in the same appointment to facilitate family trips to the healthcare center	9	96.4	Agreement	Strong
2.3	It is recommended to monitor neurodevelopment up to 6 years of age through early care centers with EOEPs (Educational and Psychopedagogical Guidance Teams), or other comprehensive neurodevelopmental assessment centers with awareness for different domains affected by Dravet syndrome, such as language, attention, executive functions, or gross and fine motor development	9	100	Agreement	Strong
2.4	In the evaluation of cognitive development, it is advisable to evaluate the communication and language of patients with Dravet syndrome at each visit with the specialist	8	82.1	Agreement	Strong
2.5	Clinical evaluation of expressive, comprehensive, and non-verbal language and its impact on learning, socialization, and safety is recommended, at least every 3–6 months	9	92.9	Agreement	Strong
2.6	Clinical evaluation of social interaction and communicative interest is recommended, at least every 3–6 months	9	89.3	Agreement	Strong
2.7	Speech evaluation is recommended, at least every 3–6 months	8	92.9	Agreement	Strong
2.8	Clinical evaluation of attention and executive functions is recommended at least every 3–6 months	8	92.9	Agreement	Strong
2.9	During the neurodevelopmental assessment, the application of measurement tools or scales quantifying the different neurodevelopmental domains should be assessed every 3–6–12 months, according to the patient's needs	9	92.8	Agreement	Strong
2.10	The neurologist and/or the pediatric neurologist, in collaboration with the neuropsychologist, should decide which scale may be more advisable to use for the evaluation of motor and cognitive development in each case, considering specific objectives and depending on the characteristics of each test	8	78.5	Agreement	Moderate
2.11	It is recommended to use the Cognitive Bayley III scale to assess different neurodevelopmental domains	7	75	Agreement	Moderate
2.12	It is recommended to use the Wechsler Intelligence scale for children to assess cognitive development	7	67.9	Agreement	Moderate
2.13	It is recommended to use the Peabody 2 Motor Development scale to assess motor development	7	71.4	Agreement	Moderate
2.14	It is recommended to use the BRIEF-2 Executive Function Behavioral Assessment scale to assess executive capabilities	7	78.6	Agreement	Moderate
2.15	It is important to assess the comorbidities or complications related to cognitive problems in Dravet syndrome, such as: Autism Spectrum Disorder (ASD), Attention Deficit and Hyperactivity Disorder (ADHD), language problems, depression, academic frustration, social isolation, and bullying	9	96.4	Agreement	Strong
2.16	Gait development should be evaluated specifically at each consultation with the specialist, at least every 3–6 months	9	85.7	Agreement	Strong
2.17	It is recommended to analyze and study ataxia and gait coordination at each consultation with the specialist, at least every 3–6 months	9	89.3	Agreement	Strong
2.18	When evaluating gait development, the use of a cart/walker or other support devices and orthoses is recommended at each visit, at least every 3–6 months	8	89.3	Agreement	Strong
2.19	It is recommended to reinforce the evaluation of motor skills and movement disorders such as parkinsonism, through assessment tools and scales, in order to obtain information systematically and periodically, at least every 6 months−1 year	8	89.3	Agreement	Strong
2.20	It is important to record videos for the evaluation of the patient's gait (by relatives or by the specialist) at each visit	8	85.7	Agreement	Strong
2.21	For the evaluation of motor development, it is recommended to quantify using specific scales with a periodicity of <1 year. Some examples are: Gross Motor Function Classification System (GMFCS) scale, Gillette scale, Pedi-CAT scale (mobility domain)	8	71.5	Agreement	Moderate
2.22	During the evaluation of motor development, it is important to consider falls, accidents and injuries at each visit, at least every 3–6 months	8	89.3	Agreement	Strong
2.23	During the evaluation of motor development, it is important to take into account muscle retraction, muscle tone and pain at each visit, at least every 3–6 months	9	85.8	Agreement	Strong
2.24	It is important to take into account orthopedic deformities in the lower limbs, as well as pathological curvatures of the spine (scoliosis, kyphoscoliosis, etc.) with an annual evaluation by Rehabilitation and Traumatology or more frequent if the clinician requires it	9	96.4	Agreement	Strong

Grey emphasis is used to highlights statements where there was no agreement or agreement was only moderate.

The panelists agreed on the importance of assessing several comorbidities or complications related to cognitive impairment. Likewise, experts agreed on recommendations related to the evaluation and study of gait and ataxia, including the frequency of evaluation.

Experts agreed on the likely suitability of a series of scales to measure neurological and motor development in patients with DS, including Bayley-III Cognitive Scale, Wechsler Intelligence Scale for Children-V, Peabody 2 Developmental Motor Scale-2, Behavior Rating Scale of Executive Function-2 (BRIEF-2), Gross Motor Function Classification System (GMFCS), Gillette Functional Assessment Questionnaire, and Pediatric Evaluation of Disability Inventory Computer Adaptive Test (PEDI-CAT). These scales should be used considering the specific objectives proposed by the adult/pediatric neurologist in collaboration with the neuropsychologist. Finally, the experts agreed on other parameters to be considered when assessing motor development, as well as on the frequency of these measurements.

### Assessment of attention and behavior in patients with DS

All the initial nine statements proposed in category 3 reached consensus agreement status (Table 3), although one (11.11%) fell into the moderate consensus category. The panelists recommended evaluating the behavior of the patient at each consultation with the specialist or at least every 3–6 months through educational/stimulation centers. According to the experts' opinion, this evaluation should consider certain comorbidities and/or situations that might be associated with the behavior. The recommended parameters for behavior evaluation included aggressiveness, impulsivity, hyperactivity, and difficulty paying attention, low cognitive flexibility, frustration intolerance, temper tantrums, family stress, and the school situation. Finally, the experts recommended assessing the use of scales on a regular basis to systematize and formalize the behavior assessment, as well as to support a possible therapeutic decision.

**Table 3:** Assessment of attention and behavior in patients with Dravet syndrome.

**Statement**		**Median**	**% Consensus**	**Result**	**Consensus level**
3.1	It is recommended to evaluate the behavior of patients with Dravet syndrome at each consultation with the specialist, at least every 3–6 months	9	92.9	Agreement	Strong
3.2	The care and behavior of the patient should be evaluated in the educational center/stimulation center at each consultation with the specialist, at least every 3–6 months	9	92.9	Agreement	Strong
3.3	When evaluating behavior, aggressiveness (heterogrevisity and self-aggressiveness) must be taken into account	9	96.4	Agreement	Strong
3.4	In assessing behavior, impulsivity, hyperactivity and difficulty paying attention must be taken into account	9	100	Agreement	Strong
3.5	In the evaluation of behavior, low cognitive flexibility, such as a poor tolerance to frustration, as well as tantrums must be taken into account	9	96.4	Agreement	Strong
3.6	When evaluating behavior, family stress and the patient's school situation should be considered	9	92.9	Agreement	Strong
3.7	For the behavioral assessment, psychiatric evaluation is recommended at least once a year; and then every 3–6 months, especially if the patient required medication	8	67.9	Agreement	Moderate
3.8	It is important to take into account certain comorbidities and/or situations associated with behavior, such as Autism Spectrum Disorder (ASD) and Attention Deficit Hyperactivity Disorder (ADHD), school failure, isolation, school exclusion and decreasing social exposure, at least every 3–6 months	9	92.9	Agreement	Strong
3.9	In order to systematize and formalize the behavior assessment, as well as to support a possible therapeutic decision, the use of scales on a regular basis (at least every 6 months or 1 year) should be assessed, such as: (A) La Child Behavior Checklist (CBCL) scale and the scale for the Assessment of Attention Deficit Hyperactivity Disorder (EDAH and ADHD-RS). (B) The scale of the Child and Adolescent Assessment System (SENA) with a psychopathological approach (anxiety, depression and hyperactivity), which can be categorized by age. (C) The Vinel and scale (to be performed by the patient's relatives) on autonomy. (D) The BRIEF 2 scale (2 versions: preschool/school). It serves both to assess executive function and behavior in relation to it	8	82.1	Agreement	Strong

Grey emphasis is used to highlights statements where there was no agreement or agreement was only moderate.

### Assessment of other comorbidities that affect the QoL in patients with DS

The panelists overall agreed on category 4 items, obtaining consensus agreement on 13 of the 14 statements (92.86%), as shown in Table 4. Two of these items (14.28%) were considered as a moderate consensus according to the panelists' opinions. The experts recommended enquiring about the patient's sleep (quantity and quality) at each visit with the specialist, at least every 3–6 months, considering the need for sleep medication and its effectiveness as well as the frequency of nighttime seizures, in particular generalized tonic-clonic seizures.

**Table 4:** Evaluation of other comorbidities that affect quality of life in patients with Dravet syndrome.

**Statement**		**Median**	**% Consensus**	**Result**	**Consensus level**
4.1	It is recommended to assess patients' sleep and quality of sleep at each visit with the specialist, at least every 3–6 months	9	96.4	Agreement	Strong
4.2	It is recommended to consider the need for medication and its effectiveness during the sleep assessment	9	92.1	Agreement	Strong
4.3	It is recommended to consider the frequency of seizures during sleep at the sleep assessment	9	100	Agreement	Strong
4.4	Regarding generalized tonic-clonic seizures during sleep, it is important to ask about them at each visit, at least every 3–6 months, and collect them independently in the seizure diaries	9	100	Agreement	Strong
4.5	Due to its relevance, it is recommended to assess the use of specific systems for the evaluation of seizures through different tools, for example: the crisis diary or applications for the collection of seizures, monitoring through video-EEG of periodically, the use of videos at home (camera in the room), the measurement of vital signs after the crisis (for example, pulse oximeter), the use of wearables and specific apps for monitoring during sleep	8	85.8	Agreement	Strong
4.6	It is recommended to take time to specifically explain to the family and/or caregivers the potential comorbidities associated with seizures during sleep. [For example: the risk of Sudden unexpected death in epilepsy -SUDEP-, daytime sleepiness (patients and relatives), the effect on marital life and the impact on social activities of the parents.]	9	89.3	Agreement	Strong
4.7	Daytime sleepiness should be specifically explored at each visit, at least every 3–6 months, in order to obtain a more complete view of the quality of the patient's nighttime sleep. For this, details must be obtained about the following aspects: the need for a specific medication, the need to measure drug plasma levels, school performance and lack of attention, secondary irritability, and sleep attacks during the day (no. naps).	9	92.9	Agreement	Strong
4.8	The use of specific scales to measure the quality of sleep of the patient and caregivers at least every 6 months should be considered, such as: the Bruni scale (sleepiness and quality of sleep in children) and the Pittsburgh scale (for adult patients, family members, and caregivers)	8	71.4	Agreement	Moderate
4.9	In cases of difficult clinical evaluation of the sleep disorder, it is recommended to complement the evaluation with the practice of an actigraphy study (wristwatch actigraph)	7	60.7	Undetermined	–
4.10	When there are symptoms of the sleep sphere, it is recommended to perform a video-EEG that includes nocturnal sleep-associated, depending on the case, with polysomnography	8	92.8	Agreement	Strong
4.11	When there are symptoms in the sleeping area, it is advisable to carry out a clinical survey directed to screen for SAHS and consider doing a PSG study	8	78.6	Agreement	Moderate
4.12	It is advisable to assess digestive comorbidity, gastroesophageal reflux, constipation at each visit, at least every 3–6 months	8	89.3	Agreement	Strong
4.13	It is advisable to assess respiratory problems and frequent infections at each visit, at least every 3–6 months	8	92.9	Agreement	Strong
4.14	It is advisable to have specific guidelines for vaccination periods and infectious processes for both family members and the doctors of the Health Center	9	96.4	Agreement	Strong

EEG, electroencephalograph; SUDEP, Sudden unexpected death in epilepsy; SAHS, sleep apnea/hypopnea syndrome; PSG, polysomnography. Grey emphasis is used to highlights statements where there was no agreement or agreement was only moderate.

Overall, the panelists agreed on various recommendations related to the assessment of sleep-related problems (including the use of the Bruni and the Pittsburgh scales), with the exception of the actigraphy study, for which consensus was undetermined. Additionally, the experts recommended spending time specifically explaining to the family and/or caregivers the potential comorbidities associated with seizures during sleep.

Experts also recommended assessing other comorbidities at least every 3–6 months, including digestive and respiratory disorders, and available specific guidelines for vaccination periods and infectious processes for both family members and the physicians of the Healthcare Center.

### Sudden unexpected death in epilepsy

Consensus was reached on all 4 items in category 5 (100%), with a strong consensus for all of them (Table 5). The panelists recommended providing information on the risk of SUDEP in DS through a specific conversation with the patient and family/caregivers. According to the experts, the pediatric/adult neurologist should lead the conversation and include prevention strategies and instructions for the management of “near-SUDEP” situations. The panelists agreed on the possibility of training caregivers on cardiopulmonary resuscitation techniques. Finally, the experts recommended carrying out an annual or biannual cardiological evaluation, based on patient risk.

**Table 5:** Sudden unexpected death in epilepsy (SUDEP).

**Statement**		**Median**	**% Consensus**	**Result**	**Consensus level**
5.1	It is advisable to inform through a specific conversation with the patient and family/caregivers about the risk of SUDEP in Dravet syndrome during the visit with the specialist	8	89.3	Agreement	Strong
5.2	It is the pediatric neurologist or neurologist who must decide the moment and the context to talk about prevention strategies and management of the patient in the case of potential “near-SUDEP” situations	8	92.9	Agreement	Strong
5.3	It is recommended to establish actions such as the possibility of carrying out a basic CPR course (Cardiopulmonary Resuscitation) in order to be prepared for an emergency situation	8	85.7	Agreement	Strong
5.4	It is recommended to carry out an annual or biannual cardiological evaluation, based on risks, including an ECG assessment of a potential long QTc and eventually a Holter ECG	8	82.1	Agreement	Strong

ECG, electrocardiogram; SUDEP, sudden unexpected death in epilepsy.

### Assessment of the QoL of caregivers/relatives

The results of the category regarding the assessment of the QoL of caregivers/relatives are shown in Table 6. Although the three items reached an agreement, only one was classified as a strong consensus (33.33%), and the other two (66.66%) fell into the moderate consensus category. The experts reached a consensus agreement on the 3 statements included in the questionnaire. The panelists recommended collecting information on the impact of the disease on the QoL of caregivers and studying the dynamics between the patient, caregivers, and siblings at least every 3–6 months. The experts recommended using quantitative assessment scales for caregiver burden and QoL, such as the Zarit Burden Interview (ZBI).

**Table 6:** Assessment of the quality of life of caregivers/relatives.

**Statement**		**Median**	**% Consensus**	**Result**	**Consensus level**
6.1	It is recommended to collect information on the impact of the disease on the quality of life of caregivers at each visit, at least every 3–6 months	8	82.1	Agreement	Strong
6.2	It is important to study the dynamics between the patient, caregivers, and siblings in order to globally analyze the situation of families and the impact it has on their quality of life and on their daily, family, social, and work activities, at least every 3–6 months	8	78.6	Agreement	Moderate
6.3	It is recommended to use quantified assessment scales for caregiver burden and quality of life, such as the Zarit scale	8	67.9	Agreement	Moderate

Grey emphasis is used to highlights statements where there was no agreement or agreement was only moderate.

## Discussion

To our knowledge, this is the first study to establish recommendations regarding QoL and PROs assessment and the main comorbidities in DS, using a Delphi technique and qualitative methodology, including caregivers and a multidisciplinary group of experts. Caregivers with a variety of familiar relationships and providing care to patients with a wide range of ages were included, and experts represented highly qualified professionals involved in the treatment and care of DS patients in Spain. The experts reached a consensus on recommendations for the management and follow-up of DS, focusing on the assessment of patients' and caregivers' QoL and other outcomes. The high degree of consensus reached by the panelists is noteworthy, with consensus agreement reached for 69 of the total 70 items proposed (Table 7), covering the six categories related to (1) general aspects of QoL evaluation of patients with DS, (2) neurodevelopmental evaluation in patients with DS, (3) assessment of attention and behavior in patients with DS, (4) evaluation of other comorbidities affecting QoL in patients with DS, (5) Sudden unexpected death in epilepsy (SUDEP), and (6) assessment of the QoL of caregivers/relatives. Full consensus agreement was obtained on the items included in all the categories, with the exception of category 4, where one item was considered undetermined. Fifteen out of the 70 items (21.43%) obtained a moderate consensus, indicating a certain level of controversy.

**Table 7:** Summary of recommendations.

It is recommended to evaluate the quality of life of the patient to determine the impact of the disease on their physical, mental and social wellbeing, facilitating follow-up, and decision-making by physicians
At each visit with the specialist, it is recommended to assess the quality of life of the patient taking into account the patient's biological or chronological age, according to the characteristics to be evaluated
It is recommended to assess the quality of life of the patient through specific questionnaires aimed at the parents or caregivers, taking into account the patient's biological or chronological age, according to the characteristics to be evaluated
In assessing quality of life, caregivers and family members should have tools such as self-administered questionnaires to be able to report the patient's situation to the specialist in a structured and concrete way
The quality of life questionnaires used to monitor the patient should include specific questions on: Aspects related to physical health status
The quality of life questionnaires used to monitor the patient should include specific questions on: Aspects related to the psychological status
The quality of life questionnaires used to monitor the patient should include specific questions on: Aspects related to the social environment and functioning, such as the family or school environment
The quality of life questionnaires used to monitor the patient should include specific questions on: Aspects related to the financial, housing conditions and supporting material
The quality of life questionnaires used for patient follow-up should include specific questions on: Aspects related to adverse effects of drug treatment
The quality of life questionnaires used to monitor the patient should include specific questions on: Aspects related to the relationship of the patient and caregivers with healthcare professionals
The quality of life questionnaires used to monitor the patient should include specific questions on: Aspects related to the relationship of the patient and caregivers with the healthcare system
The electronic format is considered the optimal method for collecting information on quality of life (with different alternatives for non-technology users, such as paper format)
The pediatrician, family doctor, pediatric neurologist, or neurologist should be the one to assess the results of the questionnaires on quality of life provided and to ask for them regularly and specifically in their visits and consultations
Patient quality of life questionnaires should always be provided and managed by healthcare professionals, with specialized nursing personnel, at the hospital or in a primary care center being the most recommended setting
It is recommended to monitor and maintain fluent and constant communication between the different professionals involved in the quality of life of the patient, their family members, and caregivers
It is recommended to assess the neurodevelopment of patients with Dravet syndrome at each visit with the specialist (every 3 months from 0 to 3 years, every 6 months from 3 to 6 years, and every 12 months from 7 years onwards)
It is recommended for all patients with Dravet syndrome to be assessed and monitored by a multidisciplinary team of neuropsychologists, speech therapists, occupational therapists and physiotherapists, grouping specialists in the same appointment to facilitate family trips to the healthcare center
It is recommended to monitor neurodevelopment up to 6 years of age through early care centers with EOEPs (Educational and Psychopedagogical Guidance Teams), or other comprehensive neurodevelopmental assessment centers with awareness for different domains affected by Dravet syndrome, such as language, attention, executive functions, or gross and fine motor development
In the evaluation of cognitive development, it is advisable to evaluate the communication and language of patients with Dravet syndrome at each visit with the specialist
Clinical evaluation of expressive, comprehensive, and non-verbal language and its impact on learning, socialization, and safety is recommended, at least every 3–6 months
Clinical evaluation of social interaction and communicative interest is recommended, at least every 3–6 months
Speech evaluation is recommended, at least every 3–6 months
Clinical evaluation of attention and executive functions is recommended at least every 3–6 months
During the neurodevelopmental assessment, the application of measurement tools or scales quantifying the different neurodevelopmental domains should be assessed every 3–6–12 months, according to the patient's needs
The neurologist and/or the pediatric neurologist, in collaboration with the neuropsychologist, should decide which scale may be more advisable to use for the evaluation of motor and cognitive development in each case, considering specific objectives and depending on the characteristics of each test
It is recommended to use the Cognitive Bayley III scale to assess different neurodevelopmental domains
It is recommended to use the Wechsler Intelligence scale for children to assess cognitive development
It is recommended to use the Peabody 2 Motor Development scale to assess motor development
It is recommended to use the BRIEF-2 Executive Function Behavioral Assessment scale to assess executive capabilities
It is recommended to evaluate the quality of life of the patient to determine the impact of the disease on their physical, mental and social wellbeing, facilitating follow-up, and decision-making by physicians
It is important to assess the comorbidities or complications related to cognitive problems in Dravet syndrome, such as: Autism Spectrum Disorder (ASD), Attention Deficit and Hyperactivity Disorder (ADHD), language problems, depression, academic frustration, social isolation, and bullying
Gait development should be evaluated specifically at each consultation with the specialist, at least every 3–6 months
It is recommended to analyze and study ataxia and gait coordination at each consultation with the specialist, at least every 3–6 months
When evaluating gait development, the use of a cart/walker or other support devices and orthoses is recommended at each visit, at least every 3–6 months
It is recommended to reinforce the evaluation of motor skills and movement disorders such as parkinsonism, through assessment tools and scales, in order to obtain information systematically and periodically, at least every 6 months-1 year
It is important to record videos for the evaluation of the patient's gait (by relatives or by the specialist) at each visit
For the evaluation of motor development, it is recommended to quantify using specific scales with a periodicity of <1 year. Some examples are: Gross Motor Function Classification System (GMFCS) scale, Gillette scale, Pedi-CAT scale (mobility domain)
During the evaluation of motor development, it is important to consider falls, accidents and injuries at each visit, at least every 3–6 months
During the evaluation of motor development, it is important to take into account muscle retraction, muscle tone and pain at each visit, at least every 3–6 months
It is important to take into account orthopedic deformities in the lower limbs, as well as pathological curvatures of the spine (scoliosis, kyphoscoliosis, etc.) with an annual evaluation by Rehabilitation and Traumatology or more frequent if the clinician requires it
It is recommended to evaluate the behavior of patients with Dravet syndrome at each consultation with the specialist, at least every 3–6 months
The care and behavior of the patient should be evaluated in the educational center/stimulation center at each consultation with the specialist, at least every 3–6 months
When evaluating behavior, aggressiveness (heterogrevisity and self-aggressiveness) must be taken into account
In assessing behavior, impulsivity, hyperactivity and difficulty paying attention must be taken into account
In the evaluation of behavior, low cognitive flexibility, such as a poor tolerance to frustration, as well as tantrums must be taken into account
When evaluating behavior, family stress and the patient's school situation should be considered
For the behavioral assessment, psychiatric evaluation is recommended at least once a year; and then every 3–6 months, especially if the patient required medication
It is important to take into account certain comorbidities and/or situations associated with behavior, such as Autism Spectrum Disorder (ASD) and Attention Deficit Hyperactivity Disorder (ADHD), school failure, isolation, school exclusion and decreasing social exposure, at least every 3–6 months
In order to systematize and formalize the behavior assessment, as well as to support a possible therapeutic decision, the use of scales on a regular basis (at least every 6 months or 1 year) should be assessed, such as: (A) La Child Behavior Checklist (CBCL) scale and the scale for the Assessment of Attention Deficit Hyperactivity Disorder (EDAH and ADHD-RS). (B) The scale of the Child and Adolescent Assessment System (SENA) with a psychopathological approach (anxiety, depression and hyperactivity), which can be categorized by age. (C) The Vinel and scale (to be performed by the patient's relatives) on autonomy. (D) The BRIEF 2 scale (2 versions: preschool/school). It serves both to assess executive function and behavior in relation to it
It is recommended to assess patients' sleep and quality of sleep at each visit with the specialist, at least every 3–6 months
It is recommended to consider the need for medication and its effectiveness during the sleep assessment
It is recommended to consider the frequency of seizures during sleep at the sleep assessment
Regarding generalized tonic-clonic seizures during sleep, it is important to ask about them at each visit, at least every 3–6 months, and collect them independently in the seizure diaries
Due to its relevance, it is recommended to assess the use of specific systems for the evaluation of seizures through different tools, for example: the crisis diary or applications for the collection of seizures, monitoring through video-EEG of periodically, the use of videos at home (camera in the room), the measurement of vital signs after the crisis (for example, pulse oximeter), the use of wearables and specific apps for monitoring during sleep
It is recommended to take time to specifically explain to the family and/or caregivers the potential comorbidities associated with seizures during sleep. (For example: the risk of Sudden unexpected death in epilepsy -SUDEP-, daytime sleepiness (patients and relatives), the effect on marital life and the impact on social activities of the parents)
Daytime sleepiness should be specifically explored at each visit, at least every 3–6 months, in order to obtain a more complete view of the quality of the patient's nighttime sleep. For this, details must be obtained about the following aspects: the need for a specific medication, the need to measure drug plasma levels, school performance and lack of attention, secondary irritability, and sleep attacks during the day (no. naps).
The use of specific scales to measure the quality of sleep of the patient and caregivers at least every 6 months should be considered, such as: the Bruni scale (sleepiness and quality of sleep in children) and the Pittsburgh scale (for adult patients, family members, and caregivers)
When there are symptoms of the sleep sphere, it is recommended to perform a video-EEG that includes nocturnal sleep-associated, depending on the case, with polysomnography
When there are symptoms in the sleeping area, it is advisable to carry out a clinical survey directed to screen for SAHS and consider doing a PSG study
It is advisable to assess digestive comorbidity, gastroesophageal reflux, constipation at each visit, at least every 3–6 months
It is advisable to assess respiratory problems and frequent infections at each visit, at least every 3–6 months
It is recommended to evaluate the quality of life of the patient to determine the impact of the disease on their physical, mental and social wellbeing, facilitating follow-up, and decision-making by physicians
It is advisable to have specific guidelines for vaccination periods and infectious processes for both family members and the doctors of the Health Center
It is advisable to inform through a specific conversation with the patient and family/caregivers about the risk of SUDEP in Dravet syndrome during the visit with the specialist
It is the pediatric neurologist or neurologist who must decide the moment and the context to talk about prevention strategies and management of the patient in the case of potential “near-SUDEP” situations
It is recommended to establish actions such as the possibility of carrying out a basic CPR course (Cardiopulmonary Resuscitation) in order to be prepared for an emergency situation
It is recommended to carry out an annual or biannual cardiological evaluation, based on risks, including an ECG assessment of a potential long QTc and eventually a Holter ECG
It is recommended to collect information on the impact of the disease on the quality of life of caregivers at each visit, at least every 3–6 months
It is important to study the dynamics between the patient, caregivers, and siblings in order to globally analyze the situation of families and the impact it has on their quality of life and on their daily, family, social, and work activities, at least every 3–6 months
It is recommended to use quantified assessment scales for caregiver burden and quality of life, such as the Zarit scale

EEG, electroencephalograph; SUDEP, Sudden unexpected death in epilepsy; SAHS, sleep apnea/hypopnea syndrome; PSG, polysomnography.

Grey denotes recommendations with moderate agreement.

Appropriate assessment of clinical and QoL parameters of patients with DS is crucial due to the importance of the symptoms and burden of the disease (14, 30, 31). In this regard, it is important to note that some treatments can negatively affect DS patients, as is the case of sodium channel blockers (SCBs) on cognition (32). Although a European clinical guideline was recently published (22), the publication focused on the treatment of DS, leaving gaps with respect to the QoL and comorbidity assessment in DS. Seizure severity, cognition, and motor and behavioral problems appear to be the primary drivers of QoL (32, 33). However, despite the available publications, clear guidelines for its routine assessment in both DS patients and caregivers are still missing (23–27). This Delphi consensus was developed to meet such needs and aimed to provide a set of recommendations for clinicians involved in the management of patients with DS.

The recommendations regarding general aspects of QoL evaluation of patients with DS indicate that experts overall consider the evaluation of the patient's QoL at each visit with the clinician to be of great importance. This opinion is supported by several publications indicating an association between clinical parameters and the QoL status of patients with DS (23, 30, 34). The experts also recommended including a wide range of parameters in the assessment of patients' QoL, considering its multidimensional nature. Although there was a consensus agreement on most of the contents of the instruments for assessing QoL, including pharmacological treatment, social, physical, and psychological aspects (35–37), a higher proportion of panelists disagreed on the assessment of the financial and home situation, support material, relationships with healthcare professionals and the healthcare system. Likewise, despite reaching a consensus agreement, several panelists disagreed on the need for questionnaires to be administered by physicians. In conclusion, the recommendations of this category are in favor of establishing the use of PROs in the clinical practice of DS. Given the infrequent use of PROs in this pathology (21), the publication of these recommendations may be a stimulus for their development and implementation.

For neurodevelopmental evaluation in patients with DS, the panelists recommended that assessments be conducted at each visit with the specialist, with varying frequencies depending on the patient's age. Given the neurodevelopmental delay in DS children (38–40), the experts considered that a frequent measurement of neurodevelopment was appropriate. Because of the multiple areas that may be affected by a neurodevelopment delay (38–40), the panelists recommended its assessment using several scales that have been previously validated in the pediatric setting (41–46), although in the case of Bayley III, Wechsler, Peabody 2, and BRIEF-2, there was less agreement on the consensus.

Regarding the assessment of attention and behavior in patients with DS, the panelists recommended evaluating the behavior of patients with DS in the educational/stimulation center at each consultation with the specialist, with a frequency of at least 3–6 months. For this statement, the percentage of consensus reached 67.9%, close to the limit of agreement, which may be due to the indicated follow-up time. Several panelists commented that a follow-up of 6 months is sufficient and more in line with the reality of clinical practice, with limited time for each patient visit. The motivation for this recommendation is that behavioral problems in DS are very common and have a tremendous impact on these patients' lives (47). Patients with DS present multiple behavioral problems and, therefore, experts recommend evaluating several parameters within this domain (47).

During the assessment of other comorbidities affecting QoL in patients with DS, a total of 13 statements of the 14 proposed obtained a consensus agreement. The only item not validated in the entire Delphi questionnaire was the one related to the use of actigraphy study for evaluating a sleep disorder. The reason for this may be the scarcity of studies on this subject conducted in the SD population (48). The remaining items on the importance of assessing sleep, as well as other comorbidities, reached consensus agreement by the experts, likely motivated by the high prevalence of other diseases in patients with DS (49–51). In this regard, the advice to assess respiratory problems may be due to the fact that seizures can increase the risk of pulmonary complications, such as aspiration pneumonia, which occurs when patients inhale food, stomach acid, or saliva into the lungs, potentially resulting in sepsis (50).

SUDEP is the leading reported cause of death reported in DS, accounting for nearly half of all premature deaths (52). The experts recommended assessing several aspects related to SUDEP in category 5. The panelists agreed that it is advisable to inform through a specific discussion with the patient and family/caregivers about the risk of SUDEP and to educate them on prevention and self-assessment strategies. Given the association between SUDEP and heart failure, periodic cardiological evaluations were recommended (52).

DS is a disease with a significant impact on patients' caregivers and relatives (53, 54). For this reason, in category 6, the experts reached a consensus to recommend collecting information regarding the impact of the disease on the QoL of caregivers and to study the dynamics between patients, caregivers, and siblings at least every 3–6 months. The ZBI was proposed as an assessment measure, presumably because it has been used in the field of epilepsy (53).

The Delphi technique is widely used in health studies as a method to obtain useful information from experts when the published body of evidence is incomplete (23, 24, 28, 55). However, the conclusions obtained with this methodology may have several limitations associated with the setting and design of this Delphi questionnaire. In the present study, the panelists were exclusively from Spain and, thus, their experience was focused on the Spanish healthcare system. Therefore, the recommendations of this study, such as those related to the frequency of assessments, may not be appropriate for other countries or regions. Likewise, this study has not assessed the impact that the implementation of these recommendations could have on the current Spanish healthcare system from the point of view of the potential need for more human and non-human resources. Clinicians with experience in other healthcare systems should assess whether each recommendation in this paper can be translated into their clinical practice, and adapt or discard those that they do not consider appropriate. Nevertheless, this study used a Delphi questionnaire developed by experts with the input from patient caregivers, which allowed for capturing controversial issues from the experts' and caregivers' perspectives. Another possible limitation of the study relates to the limited number of caregivers who participated in the focus group. Although the intention was to make it as representative and diverse as possible, it is possible that some bias could become apparent at some points, given that not all the perspectives of caregivers of DS patients may have been represented. In addition, further studies involving professionals from a wider range of disciplines involved in the care and management of patients with DS, such as social workers or therapists, may provide a more accurate view on the management of PROs in the daily life of DS patients and their families. Another limitation of the study associated to the Delphi technique is related to the consensus definition, which is not standard, and therefore, different criteria can be found in the literature (23, 29). For this reason, we deemed appropriate to define two categories of consensus, moderate and strong (29). We considered that although agreement was reached on most of the items consulted, those that fell into the moderate consensus category may have raised more controversy among the experts.

The high burden on QoL for DS patients and their caregivers, as well as the lack of concise guidelines on the evaluation of various clinical parameters, makes it necessary to issue expert recommendations to fill these gaps. The present study intended to meet these needs through an expert consensus, which may be a useful guide for clinicians involved in the routine follow-up and management of patients with DS. In particular, this study focused on PROs, which constitute a patient-centered alternative to incorporate into future clinical practice of patients with DS, as classical measures fail to cover all aspects of the disease (56). Furthermore, this study may serve as an example for the development of recommendations to assess PROs and other outcomes in other DEEs.

## Data availability statement

The original contributions presented in the study are included in the article/Supplementary material, further inquiries can be directed to the corresponding author.

## Ethics statement

Ethical review and approval was not required for the study on human participants in accordance with the local legislation and institutional requirements. Written informed consent from the patients/participants or patients/participants' legal guardian/next of kin was not required to participate in this study in accordance with the national legislation and the institutional requirements.

## The Charlotte Project group

Adrián García-Ron, Alberto Vieco, Alfonso Amado Puentes, Helena Alarcón Martínez, Irene García Morales, Javier Aparicio Calvo, Jesús Eirís Puñal, Juan Jesús Rodriguez Uranga, Juan José Poza Aldea, Julián Lara Herguedas, Julio Ramos-Lizana, Mercè Falip Centelles, Mercedes Garcés Sánchez, Miquel Raspall Chaure, Patricia Smeyers Dura, Rafael Toledano Delgado, Rocío Calvo Medina, Salvador Ibáñez Micó, Sergio Aguilera Albesa, Víctor Soto Insuga, and Xiana Rodríguez Osorio.

## Author contributions

ÁA-S has contributed to the design and conception of the study. All authors have contributed to the acquisition, analysis, interpretation of the data, have participated in the drafting and reviewing of the manuscript, and approving the submitted version.

## Funding

This study was funded and promoted by Zogenix Spain, now a part of UCB.

## Conflict of interest

Author ÁA-S has received funding for educational and research activities from Angelini, Zogenix, UCB, PTC pharma, Blueprint genetics, GW, Eisai. Author AG-N has received advisory or research funds by Arvelle/Angelini, Bial, Biocodex, EISAI, Esteve, GW Pharma, Jazz Pharmaceuticals, PTC Therapeutics, Stoke, UCB Pharma and Zogenix. Author JA is president of the Dravet Syndrome Foundation Spain (DSF). He and/or the DSF have received grants and/or financial support from GW Pharma, Zogenix, Ovid Therapeutics, Encoded Therapeutics, Biocodex, Praxis, Health in Code, Takeda, UCB, Epygenix, Jazz Pharmaceuticals and StrideBio to help carry out some of the DSF's foundational activities or provide consulting services. Author JA honoraria have always been donated directly or indirectly to the DSF. Author RS-C has been a trial investigator for GW Pharmaceuticals, Takeda Pharmaceutical Company Ltd. Author and Zogenix and has served on advisory boards for Novartis, GW Pharmaceuticals, Biocodex and Zogenix. Author JG-P has received advisory or lecture funds by BIAL, EISAI, GW, NUTRICIA, SANOFI, UCB and ZOGENIX. VV has participated in advisory boards and symposia organized by Angellini, Bial, Eisai Inc., GW pharma, Novartis, Takeda, UCB, Zogenix. Author AM was employed by Dracaena Consulting SL. Author LA was employed by Apoyo Dravet. The remaining authors declare that the research was conducted in the absence of any commercial or financial relationships that could be construed as a potential conflict of interest.

## Publisher's note

All claims expressed in this article are solely those of the authors and do not necessarily represent those of their affiliated organizations, or those of the publisher, the editors and the reviewers. Any product that may be evaluated in this article, or claim that may be made by its manufacturer, is not guaranteed or endorsed by the publisher.
