# Supplementary material for: The Charlotte Project: Recommendations for patient-reported outcomes and clinical parameters in Dravet syndrome through a qualitative and Delphi consensus study
Source: Front Neurol. 2022 Sep 1;13:975034. doi: 10.3389/fneur.2022.975034 (PMC9481303; doi:10.3389/fneur.2022.975034)
Supplement: Supplementary file 1 [file Table_1.docx]

| **Supplementary Table 1.** Caregivers’ relationship with patient an patients’ age | |
| --- | --- |
| **Caregiver** | **Patients’ age (years)** |
| Mother | 1 |
| Father | 4 |
| Mother | 7 |
| Father | 10 |
| Father | 13 |
| Father | 16 |
| Mother | 33 |
| Sister | 50 |
