# Supplementary material for: The Charlotte Project: Recommendations for patient-reported outcomes and clinical parameters in Dravet syndrome through a qualitative and Delphi consensus study
Source: Front Neurol. 2022 Sep 1;13:975034. doi: 10.3389/fneur.2022.975034 (PMC9481303; doi:10.3389/fneur.2022.975034)
Supplement: Supplementary file 2 [file Table_2.docx]

**Supplementary Table 2.** Caregivers’ relationship with patient an patients’ age

| **BLOCK I. Aspects related to the importance of the quality of life.**   1. In relation to general aspects of quality of life, do you usually ask about these aspects in the healthcare setting? 2. In reference to questions about quality of life issues, to what extent do you think it is important to ask about them in the healthcare setting? 3. Who do you think should provide the questionnaires on aspects of quality of life? 4. In your experience, how important do you think it is to be asked about the following aspects in relation to quality of life?    1. Aspects related to physical fitness (e.g. strength, endurance, flexibility, quality of sleep...).    2. Aspects related to psychological state.    3. Aspects related to the social sphere.    4. Aspects related to the side effects of disease treatment.    5. Aspects related to the relationship with the specialist physician. 5. In your opinion, where do you think it should be asked about these quality of life issues? 6. In reference to the way of collecting information on quality of life, through which format do you think its collection should be carried out? 7. Regarding the use of these quality of life data by the specialist following Dravet syndrome, how important do you value the use of these? 8. How often do you think quality of life data should be evaluated by the Dravet syndrome follow-up specialist? 9. Do you consider other important aspects related to quality of life that have not been mentioned? |
| --- |
| **BLOCK II. Impact of comorbidities on patient's quality of life.**   1. Rate from 1 to 5 (1 does not affect-5 affects a lot), how each of the following aspects related to neurodevelopment affects the patient's quality of life:    1. Attention and ability to concentrate.    2. Learning process.    3. Communication and language.    4. Playing.    5. Gait and walking ability.    6. Hand skills. 2. Rate from 1 to 5 (1 does not affect-5 affects a lot), how each of the following behavioral aspects affects the patient's quality of life:    1. General behavior at home.    2. Behavior in educational center/stimulation center.    3. Social behavior in leisure environments (meetings with friends, family, etc.).    4. Respect for limits and rules.    5. Aggressiveness.    6. Adaptability to change.    7. Self-support.    8. Other related aspects. Intestinal habits (diarrhea, etc.).    9. Other related aspects. Food and appetite. 3. Rate from 1 to 5 (1 does not affect-5 affects a lot), how each of the following aspects related to sleep affects the patient's quality of life:    1. Difficulty in falling asleep.    2. Awakenings.    3. Seizures during sleep.    4. Daytime sleepiness.    5. Regular sleep rhythms. |
| **BLOCK III. Impact of dravet syndrome on the caregivers' quality of life.**   1. Rate from 1 to 5 (1 does not affect-5 affects a lot), how each of the following aspects affects the quality of life of the caregivers:    1. Emotional impact (e.g. mood, stress, fear, worry, frustration...).    2. Physical impact (For example: fatigue, headache, cervical and lumbar pain, etc.).    3. Impact on work and the economy (difficulty to work, absence or sick leave...).    4. Impact on daily activities (e.g. constant care, interruptions...).    5. Impact on leisure (For example: in leisure activities, free time ...).    6. Impact on social life (e.g. relationship with other people...).    7. Impact on sleep (e.g. insomnia, sleep interruption...).    8. Need for medication (medication for pain, insomnia, mood, anxiety...). 2. Do you consider other important aspects related to the quality of life of the patient and the caregiver that have not been mentioned? |
