# Supplementary material for: The Charlotte Project: Recommendations for patient-reported outcomes and clinical parameters in Dravet syndrome through a qualitative and Delphi consensus study
Source: Front Neurol. 2022 Sep 1;13:975034. doi: 10.3389/fneur.2022.975034 (PMC9481303; doi:10.3389/fneur.2022.975034)
Supplement: Supplementary file 3 [file Table_3.docx]

| **Supplementary Table 3.** Characteristics of the panel experts | |
| --- | --- |
| **Gender,** *n (%)* |  |
| Female | 10 (35.71) |
| Male | 18 (64.28) |
| **Age,** mean (SD) | 47.64 (7.23) |
| **Medical specialties,** n (%) |  |
| Neurology | 15 (53.57) |
| Pediatric neurology | 13 (46.43) |
| **Region,** n (%) |  |
| Andalucía | 4 (14.28) |
| Cataluña | 4 (14.28) |
| Comunidad Foral de Navarra | 2 (7.14) |
| Comunidad de Madrid | 9 (32.14) |
| Comunidad Valenciana | 3 (10.71) |
| Galicia | 3 (10.71) |
| País Vasco | 1 (3.57) |
| Región de Murcia | 2 (7.14) |
