# Supplementary figures and images for: The Charlotte Project: Recommendations for patient-reported outcomes and clinical parameters in Dravet syndrome through a qualitative and Delphi consensus study
Source: Front Neurol. 2022 Sep 1;13:975034. doi: 10.3389/fneur.2022.975034 (PMC9481303; doi:10.3389/fneur.2022.975034)

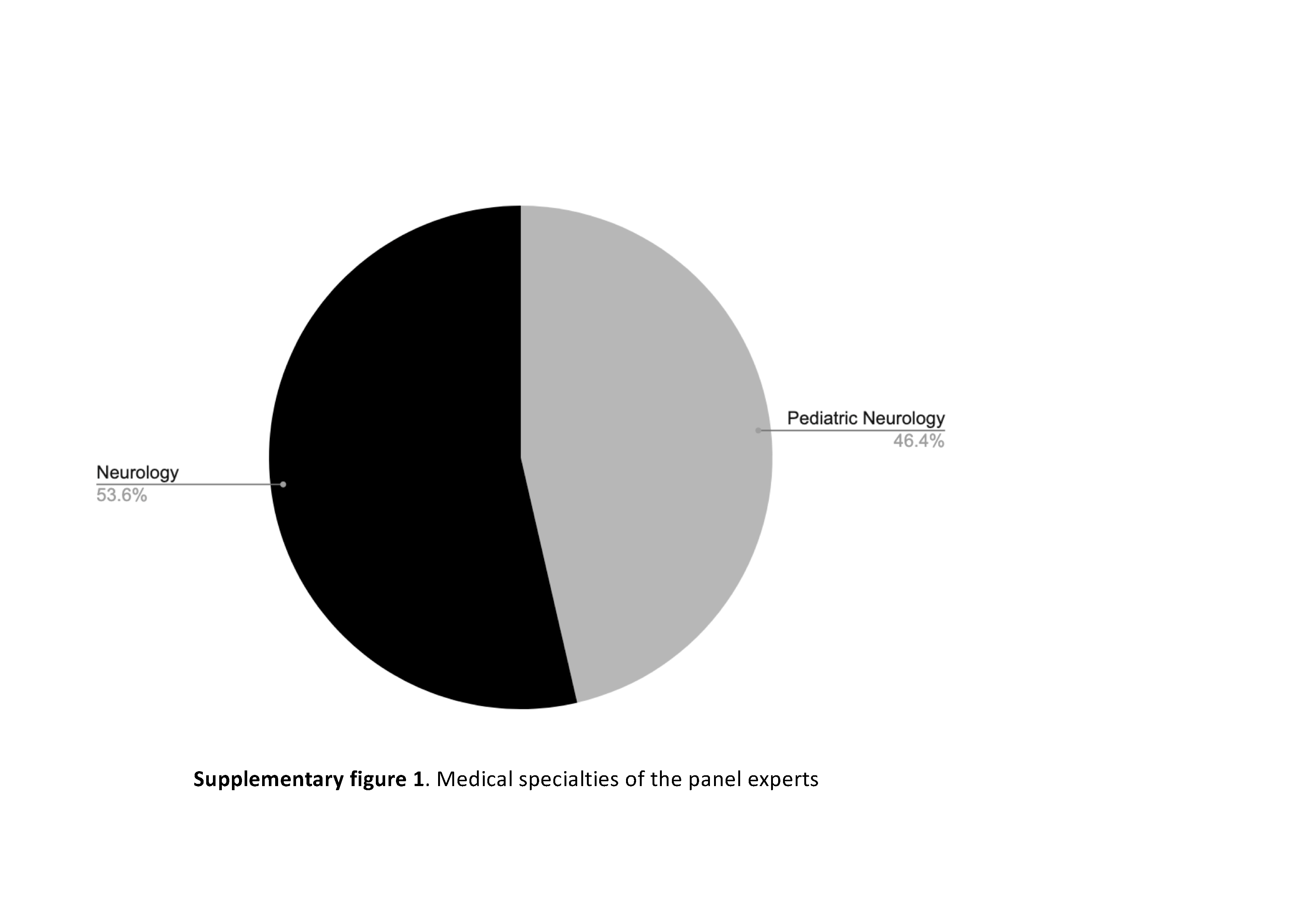

Supplement: Supplementary file 4 [file Image_1.TIF]
